# Phenylketonuria Diet Promotes Shifts in Firmicutes Populations

**DOI:** 10.3389/fcimb.2019.00101

**Published:** 2019-04-16

**Authors:** Giulia Bassanini, Camilla Ceccarani, Francesca Borgo, Marco Severgnini, Valentina Rovelli, Giulia Morace, Elvira Verduci, Elisa Borghi

**Affiliations:** ^1^Department of Health Sciences, Università degli Studi di Milano, Milan, Italy; ^2^Institute of Biomedical Technologies, National Research Council, Segrate, Italy; ^3^Department of Pediatrics, San Paolo Hospital, Università degli Studi di Milano, Milan, Italy

**Keywords:** phenylketonuria, mild hyperphenylalaninemia, diet, microbiota, glycemic index, *Faecalibacterium prausnitzii*, butyrate

## Abstract

Low-phenylalanine diet, the mainstay of treatment for phenylketonuria (PKU), has been shown to increase glycemic index and glycemic load, affecting the availability of substrates for microbial fermentation. Indeed, changes in the PKU gut microbiota compared with healthy controls have been previously reported. In this study we compared the gut microbial communities of children with PKU and with mild hyperphenylalaninemia (MHP, unrestricted diet). For each group, we enrolled 21 children (4–18 years old), for a total dataset of 42 subjects. We assessed dietary intake and performed gut microbiota analysis by sequencing the V3–V4 hypervariable regions of the 16S rRNA gene. Short chain fatty acids (SCFAs) were quantified by gas chromatographic analysis. While alpha-diversity analysis showed no significant differences between PKU and MHP groups, microbial community analysis highlighted a significant separation of the gut microbiota according to both unweighted (*p* = 0.008) and weighted Unifrac distances (*p* = 0.033). Major differences were seen within the Firmicutes phylum. Indeed, PKU children were depleted in *Faecalibacterium* spp. and enriched in *Blautia* spp. and *Clostridium* spp (family *Lachnospiraceae*). We found a divergent response of members of the Firmicutes phylum with respect to daily glycemic index, higher in PKU children. *Faecalibacterium prausnitzii*, unclassified *Ruminococcaceae* and, to a lesser extent *Roseburia* spp. negatively correlated with glycemic index, whereas unclassified *Lachnospiraceae* were positively associated. Indicator species analysis suggested *F. prausnitzii* be related to MHP status and *Ruminococcus bromii* to be associated with PKU. Despite PKU children having a higher vegetable and fiber intake, resembling a vegan diet, their gut microbial profile is different from the microbiota reported in the literature for individuals consuming a high-fiber/low-protein diet. Indeed, beneficial microorganisms, such as *F. prausnitzii*, considered a biomarker for a healthy status and one of the main butyrate producers, are depleted in PKU gut microbiota. We suggest that both the quality and quantity of carbohydrates ingested participate in determining the observed Firmicutes shifts on the PKU population.

## Introduction

Phenylketonuria (PKU; OMIM 261600) is an inherited metabolic disorder caused by a mutation in the phenylalanine hydroxylase enzyme (PAH), which converts phenylalanine (Phe) into tyrosine. As PAH activity is hampered, phenylalanine accumulates in the blood and becomes toxic to the brain (Williams et al., 2008). Allelic heterogeneity at PAH locus results in a variety of metabolic phenotypes, ranging from mild, moderate and classical PKU (blood Phe levels >360 μmol/L) to mild hyperphenylalaninemia (MHP, blood Phe levels ranging 120–360 μmol/L; Güttler and Guldberg, 1996).

Untreated PKU leads to neurodevelopmental damage and behavioral problems that are preventable by early diagnosis and dietary treatment (van Spronsen et al., 2017).

A PKU diet, started in the neonatal period and followed life-long, is characterized by low-protein natural foods (vegetables, fruits) and special low-protein products, which are low-protein variants of some foods (bread, pasta, and biscuits; Giovannini et al., 2012). Adequate protein intake is guaranteed by Phe-free amino acid mixtures (AAM) with a balanced content of amino acids and micronutrients. Despite improvements in taste, the palatability of such formulas is still less than optimal, often resulting in poor acceptance by school-aged patients. Moreover, a PKU diet has been shown to increase glycemic index and glycemic load (Moretti et al., 2017), probably due to special low-protein products frequently being enriched in sugars.

Considering the crucial role of diet in shaping the gut microbiota, i.e., the microbial community inhabiting gastrointestinal tract (Albenberg and Wu, 2014), it is not surprising that such a peculiar diet leads to microbial changes in phenylketonuric patients (Pinheiro de Oliveira et al., 2016; Verduci et al., 2018). Alterations in the gut microbiota, in turn, may influence gastrointestinal homeostasis, predispose to chronic inflammation and modulate other metabolic functions through gut-liver axis and gut-brain axis (Nieuwdorp et al., 2014).

Up to date, only a study by Pinheiro de Oliveira et al. (2016) has investigated by 16S rRNA sequencing the gut community of PKU patients. However, the small cohort (eight patients vs. ten healthy controls) and the presence of confounding factors (i.e., antibiotic treatment, age <1 year) might have mitigated the observed microbial alterations within the gut. Moreover, whether reported changes in the gut microbiota represent an effect of the disease itself or a consequence of the modified diet is still unclear.

To this end, this work aims at elucidating, by comparing the microbiota of PKU children with mild hyperphenylalaninemia, the role of the low-Phe diet as potential inducer of microbial dysbiosis.

## Materials and Methods

### Subject Recruitment and Sampling

A total of 42 children (21 PKU/21 MHP) were enrolled in the study at the Pediatric Department of San Paolo Hospital in Milan, Italy (Verduci et al., 2018). Inclusion criteria were: gestational age 37–42 week inclusive, Caucasian, living in Northern Italy, birth weight ≥2,500 g, single birth, diagnosis of PKU or MHP due to PAH deficiency. Exclusion criteria were: congenital malformation, endocrine disorders, chronic liver diseases, chronic or acute intestinal diseases, treatments with antibiotic, and probiotic/prebiotic (including glycomacropeptide) in the 3 months preceding the study. All PKU subjects started the diet therapy at disease diagnosis, usually within 10 days from birth.

Blood phenylalanine concentration was monthly monitored by the Guthrie test (Guthrie and Susi, 1963).

From all subjects we collected: anthropometric data (height, weight and *z*-score body mass index), dietary habits and stool samples, stored at −80°C until use. Three-days food diaries were filled out by a parent for each enrolled subject and processed by dieticians to calculate the average amounts of energy and nutrient intake (carbohydrates, soluble and insoluble fibers, lipids, proteins) using a commercially available software (MetaDieta®, Software version 3.1, ME.TE.DA S.r.l., San Benedetto del Tronto, Italy). For each meal, the glycemic index (GI) value and glycemic load (GL) were calculated as described by Verduci et al. (2018) using the following formulas:

GI_meal_ = (∑ _i = 1, …, n_ GI_foodi_^*^ grams of carbohydrates_foodi_)/total grams of carbohydrates_meal_

GI_daily_ = (∑ _i = 1, …, n_ GI_meali_^*^ grams of carbohydrates_meali_)/daily total grams of carbohydrates

GL_meal_ = ∑ _i = 1, …, n_ GL_foodi_.

### Gut Microbiota Analysis

Fecal DNA extraction was performed using the Spin stool DNA kit (Stratec Molecular, Berlin, Germany), according to manufacturer's instructions. For each sample, 25 ng of extracted DNA was used to construct the sequencing library. The V3–V4 hypervariable regions of the bacterial 16S rRNA were amplified with a two-step barcoding approach according to the Illumina 16S Metagenomic Sequencing Library Preparation (Illumina, San Diego, CA, USA). For library preparation, DNA samples were amplified with dual-index primers using a Nextera XT DNA Library Preparation Kit (Illumina). Library concentration and quantification were determined using a KAPA Library Quantification Kit (Kapa Biosystems, Woburn, MA, USA) and Agilent 2100 Bioanalyzer System (Agilent, Santa Clara, CA, USA), respectively. The libraries were pooled and sequenced with a MiSeq platform (Illumina) for 2 × 250 base paired-end reads and a total of 2.5 Gbases raw reads were obtained.

### Fecal Metabolite Measurement

We performed short chain fatty acids (SCFAs) and calprotectin quantification from stool samples.

Concentrations of acetic, propionic, iso-butyric, butyric, and iso-valeric acids were assessed by gas liquid chromatography in accordance with the method proposed by Weaver et al. (1989) with slight modifications described in Borgo et al. (2017). Analyses were performed using a Varian model 3400 CX Gas-chromatograph fitted with FID detector, split/splitless injector and a SPB-1 capillary column (30 m × 0.32 mm ID, 0.25 μm film thickness; Supelco, Bellefonte, PA, USA). Results are expressed as mg/g of wet weight of feces. Quantification of the SCFAs was obtained through calibration curves of acetic, propionic, iso-butyric, butyric, and iso-valeric acid in concentrations between 0.25 and 10 mM (10 mM 2-ethylbutyric acid as internal standard). SCFA data on the same cohort have been previously described in Verduci et al. (2018).

Fecal calprotectin concentrations were measured by a commercial ELISA kit (Calprotectin ELISA Kit, Immundiagnostik, Bensheim, Germany), according to manufacturer instructions.

### Absolute Quantification of *Methanobrevibacter smithii*

Real-time PCR was carried out using a SYBRGreen chemistry (ThermoScientific, USA) and the specific primers for *Methanobrevibacter smithii* (MSfw: 5′-CCGGGTATCTAATCCGGTTC-3′ and MSrev: 5′-CTCCCAGGGTAGAGGTGAAA-3′), as previously described (Borgo et al., 2017). The following thermal cycling parameters were used for amplification of DNA: 95°C for 10 min followed by 40 cycles of 15 s at 95°C, 30 s at 60°C, and 30 s at 72°C. A melting curve analysis was also performed to verify amplicon specificity.

The control strain *M. smithii* DSM-861 (DSM: Deutsche Sammlung von Mikroorganismen und Zellkulturen, Braunschweig, Germany) was used for the standard curve.

### Microbiota Profiling

The 16S rRNA gene paired sequences obtained were merged using Pandaseq (release 2.5; Masella et al., 2012), then reads were filtered by trimming stretches of 3 or more low-quality bases (quality <3) and discarding the trimmed sequences whenever they were shorter than 75% of the original one. Bioinformatic analyses were conducted using the QIIME pipeline (release 1.8.0; Caporaso et al., 2010), clustering filtered reads into Operational Taxonomic Unit (OTUs) at 97% identity level and discarding singletons (i.e., OTUs having only 1 supporting read along the whole dataset) as possible chimeras. Taxonomic assignment was performed via the RDP classifier (Wang et al., 2007) against the Greengenes database (ftp://greengenes.microbio.me/greengenes_release/gg_13_8_otus).

Indicator species analysis was performed using the indicspecies package in R (De Cáceres and Legendre, 2009) on the QIIME-derived OTU table.

Alpha-diversity was computed using the Chao1, number of OTUs, Shannon diversity, and Faith's Phylogenetic Diversity whole tree (PD whole tree) metrics; statistical evaluation among alpha-diversity indices was performed by a non-parametric Monte Carlo-based test, using 9999 random permutations. Weighted and unweighted UniFrac distances and PERMANOVA (adonis function) in the R package vegan (version 2.0-10; Oksanen et al., 2013) were used to compare the microbial community structure of the PKU and MHP children.

### Statistical Analysis

Statistical comparisons were performed using MATLAB software (Natick, MA, USA). Comparisons of the two groups were performed using Student's *t*-test for normally distributed variables and Wilcoxon test for non-normally distributed variables. For evaluating differences in relative abundances of bacterial groups, a Mann-Whitney *U*-test was performed. For each phylogenetic level, only the 25 most abundant taxa were considered, in order to focus on the major players of the gut microbiota. Due to multiple testing, a Benjamini-Hochberg correction was applied, considering a FDR < 0.15 as significant. For clarity, uncorrected *p*-values were reported in the text. Co-abundance of microbial groups, as well as correlations between taxa and nutritional values and SCFA quantities were assessed through Spearman correlation and the associated linear regression model. Unless otherwise stated, *p*-values < 0.05 were considered as significant.

## Results

### Cohort Description

Cohort characteristics are reported in Table 1. At recruitment, blood Phe levels in PKU children was slightly higher than the MHP group (*p* = 0.24). Compared with MHP children, PKU children showed higher dietary intakes of carbohydrates and fibers and a significant lower consume of proteins (expressed as %). Glycemic index (GI) and glycemic load (GL), evaluated for each meal, were significantly higher in PKU children compared with MHP subjects (*p* < 0.001). Anthropometric measurements were similar, with a BMI *z*-score not significantly different in the two groups (*p* = 0.31).

**Table 1:** Cohort characteristics and dietary habits.

	**MHP (*n* = 21)**	**PKU (*n* = 21)**	***p*-value**	
Female	12	11		
Male	9	10		
Age (years)	8.0 ± 3.4	10.0 ± 3.5	0.060	
BMI z-score	0.2 ± 1.0	0.5 ± 1.1	0.310	
Blood Phe levels (mmol/L)	228.0 ± 87.1	262.6 ± 97.5	0.241	
Carbohydrate (% energy)	56.0 ± 5.9	61.0 ± 7.0	0.047	^*^
Fiber (overall grams)	8.9 ± 2.6	16.0 ± 9.1	0.003	^**^
Protein (% energy)	52.1 ± 11.1	43.2 ± 15.1	0.023	^*^
Lipids (% energy)	32.6 ± 4.7	29.6 ± 6.6	0.188	
Glycemic index (GI)	52.8 ± 3.8	65.1 ± 5.2	<0.001	^***^
Glycemic load (GL)	104.1 ± 29.8	163.5 ± 48.6	<0.001	^***^

Values are expressed as mean ± standard deviation; one asterisk

(*) indicates p-value smaller than 0.05 (p < 0.05), two asterisks

(**) indicate p-value smaller than 0.01 (p < 0.01), three asterisks

(***) * indicate p-value smaller than 0.001 (p < 0.001; Mann-Whitney U-test)*.

### Gut Microbiota Composition in PKU and MHP Children

Five samples (2 PKU and 3 MHP subjects) were excluded from the analysis due to very low raw read quantities (with an average of 189 reads compared to an average of 159,914 reads among the other samples); the final dataset for microbiota analysis, then, consisted of 37 subjects: 19 PKU and 18 MHP.

To avoid biases related to uneven sequencing depth, samples were subsampled to 50,000 reads each. After quality filtering processes, we obtained a mean count of 49,749 ± 111 reads per sample.

**Figure 1:**
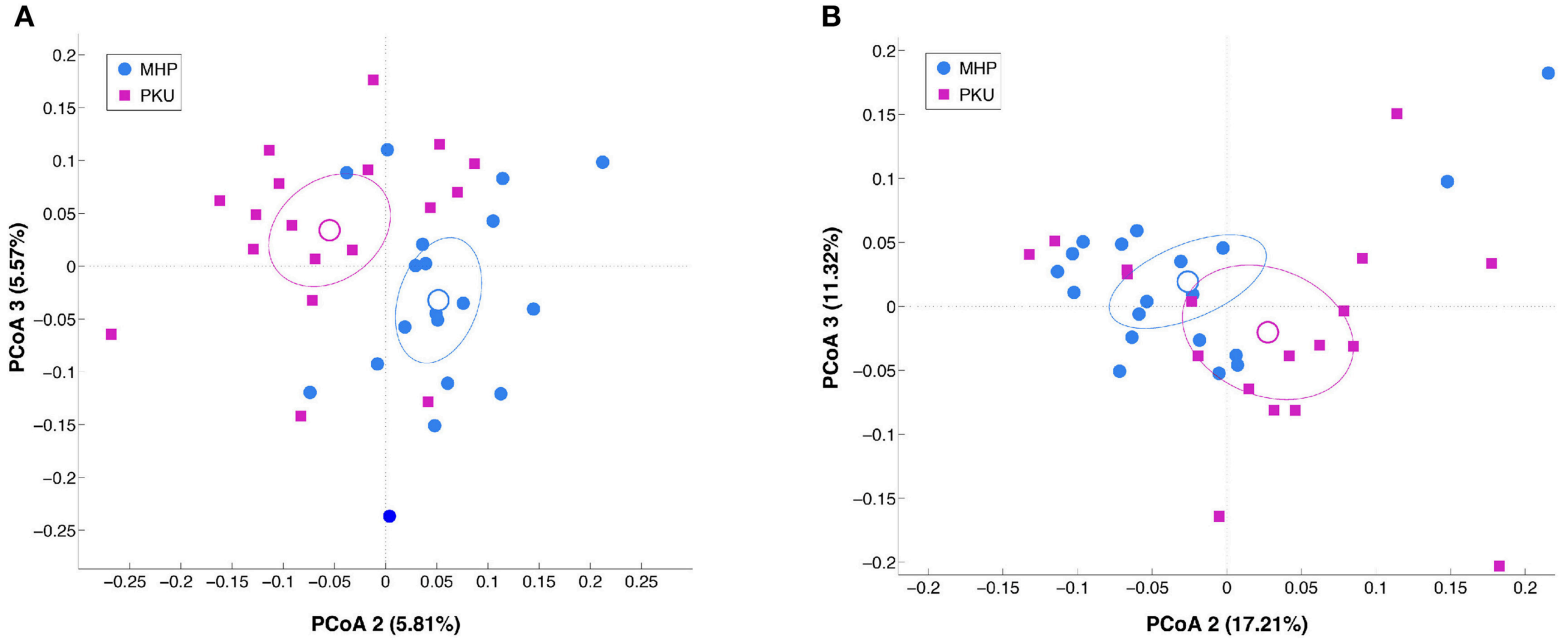
Beta-diversity analysis in MHP (blue) and PKU (magenta). Principal coordinates analysis (PCoA) of **(A)** unweighted and **(B)** weighted Unifrac distances. Microbial communities are statistically different (*adonis* test: unweighted *p* = 0.008, *R*^2^ = 0.040; weighted *p* = 0.033, *R*^2^ = 0.058). Second and third principal coordinates are shown in the plot for both distances.

Alpha-diversity analysis (data not shown) revealed no significant differences between PKU and MHP groups for any of the metrics used (number of OTUs, *p* = 0.306; chao1, *p* = 0.131; Shannon, *p* = 0.894; PD whole tree, *p* = 0.31). Beta-diversity analysis, instead, showed that the structure of the PKU fecal microbiota differed significantly from that of the MHP group according to both unweighted (*p* = 0.008) and weighted (*p* = 0.032) Unifrac distances (β-diversity, Figure 1).

**Figure 2:**
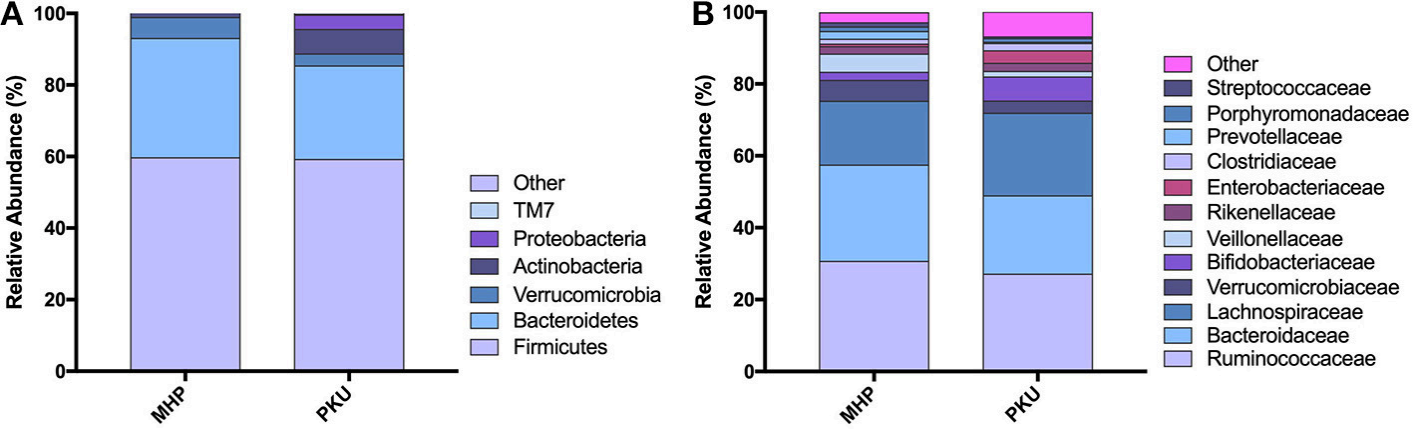
Gut microbiota composition in MHP and PKU children. Relative abundance at **(A)** phylum and **(B)** family level. All bacterial taxa present at <1% relative abundance were grouped into the “Other” classification.

The gut microbiota composition at the phylum and family levels is depicted in Figure 2 and in Table S1. The most relatively abundant phyla in PKU and MHP subjects were Firmicutes and Bacteroidetes, the latter slightly higher in MHP subjects. Among the most relatively abundant families, we found *Veillonellaceae* to be significantly depleted in PKU children (*p* = 0.002). Although not statistically significant, *Ruminococcaceae* were enriched in MHP and *Lachnospiraceae* in PKU subjects.

**Figure 3:**
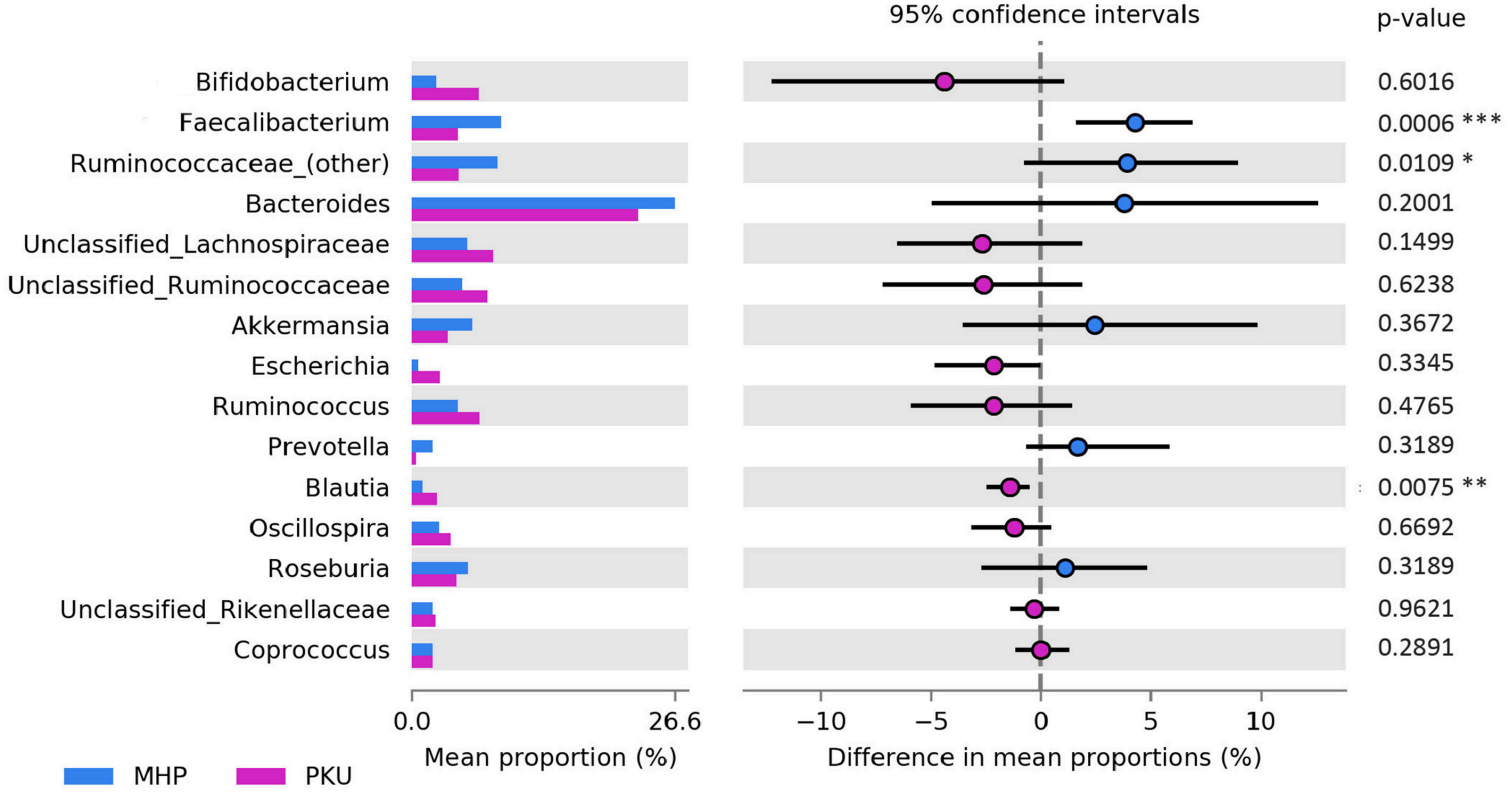
Bacterial abundances at genus level. Genera abundances of the 15 most abundant genera are reported as mean proportion along with significant *p*-values (two-sided White's non-parametric *t*-test) and 95% of confidence interval (bootstrap method).

At the genus level (Figure 3), *Faecalibacterium* (*p* = 0.001), *Ruminococcaceae (other)* (*p* = 0.03) were more relatively abundant in MHP children; although not significant, *Bacteroides* and *Prevotella* genera showed the same trend. Furthermore, we observed an increased *Prevotella/Bacteroides* ratio in MHP compared with PKU (0.14 ± 0.59; 0.02 ± 0.05, respectively).

In contrast, PKU children were characterized by a significant increase in the relative abundance of *Blautia* (*p* = 0.004), *Clostridium* (belonging to *Lachnospiraceae* family, *p* = 0.002), and *Lachnospiraceae (other)* (*p* = 0.019). Significantly altered taxa belonged to the Firmicutes phylum and are highlighted in Table 2.

**Table 2:** Genera belonging to Firmicutes phylum significantly increased or depleted in PKU children (Mann-Whitney *U*-test, *p-value* < 0.05).

**Genus**	**MHP (mean ± SD)**	**PKU (mean ± SD)**	***p*-value**	**PKU**
*Ruminococacceae* (other)	7.07 ± 8.26	2.78 ± 4.22	0.030	**↓**
*Lachnospiraceae* (other)	0.35 ± 0.25	1.84 ± 2.52	0.019	**↑**
*Blautia*	1.07 ± 0.85	2.43 ± 2.02	0.004	**↑**
*Faecalibacterium*	8.71 ± 4.32	4.52 ± 4.14	0.001	**↓**
*Clostridium*	0.23 ± 0.29	1.92 ± 2.64	0.002	**↑**
*Dialister*	3.28 ± 3.83	0.67 ± 2.1	0.036	**↓**

Because of the underestimation of Archaea by 16S rRNA gene sequencing, Real-time PCR quantification of the most abundant Archaea species in human gut microbiota, *Methanobrevibacter smithii*, was performed. We did not observe significant differences between PKU and MHP subjects (*p* = 0.40).

Indicator species analysis highlighted *Akkermansia muciniphila* OTU 1045 (best BLAST hit: Accession number NR_074436.1, with 97% seq. similarity over 420 bp, e*-value* = 0.046) and *Faecalibacterium prausnitzii* OTU 3793 (accession number: NR_028961.1, with 97% seq. similarity over 420 bp, e*-value* = 0.001) to be characteristic of MHP microbiota, whereas *Ruminococcus bromii* OTU 3232 (accession number: NR_025930.1, with 97% seq. similarity over 420 bp, e*-value* = 0.018) to be associated to PKU.

### Bacterial Correlation Patterns

Because cross-feeding between species is relevant in gut microbiota dynamic, we performed correlation analysis to study interactions between different members of intestinal microbiota. Several significant bacterial connections (Table S2), both positive and negative, have been observed. In particular, MHP indicator species *A. muciniphila* and *F. prausnitzii* showed several interactions with other members of the microbial community: *Akkermansia* was negatively related to *Unclassified Lachnospiraceae* (*R* = −0.61) and to *Blautia* spp. (*R* = −0.40), while was positively associated to *Oscillospira* (*R* = 0.57) and to *Unclassified Clostridiales* (*R* = 0.34). On the other hand, *F. prausnitzii* was only found positively related to *Ruminococcaceae* (other) genus (*R* = 0.42). The PKU indicator species, *Ruminococcus bromii*, instead, showed no correlation to other genera itself.

### Correlations Between Gut Microbiota and Nutritional Values

**Figure 4:**
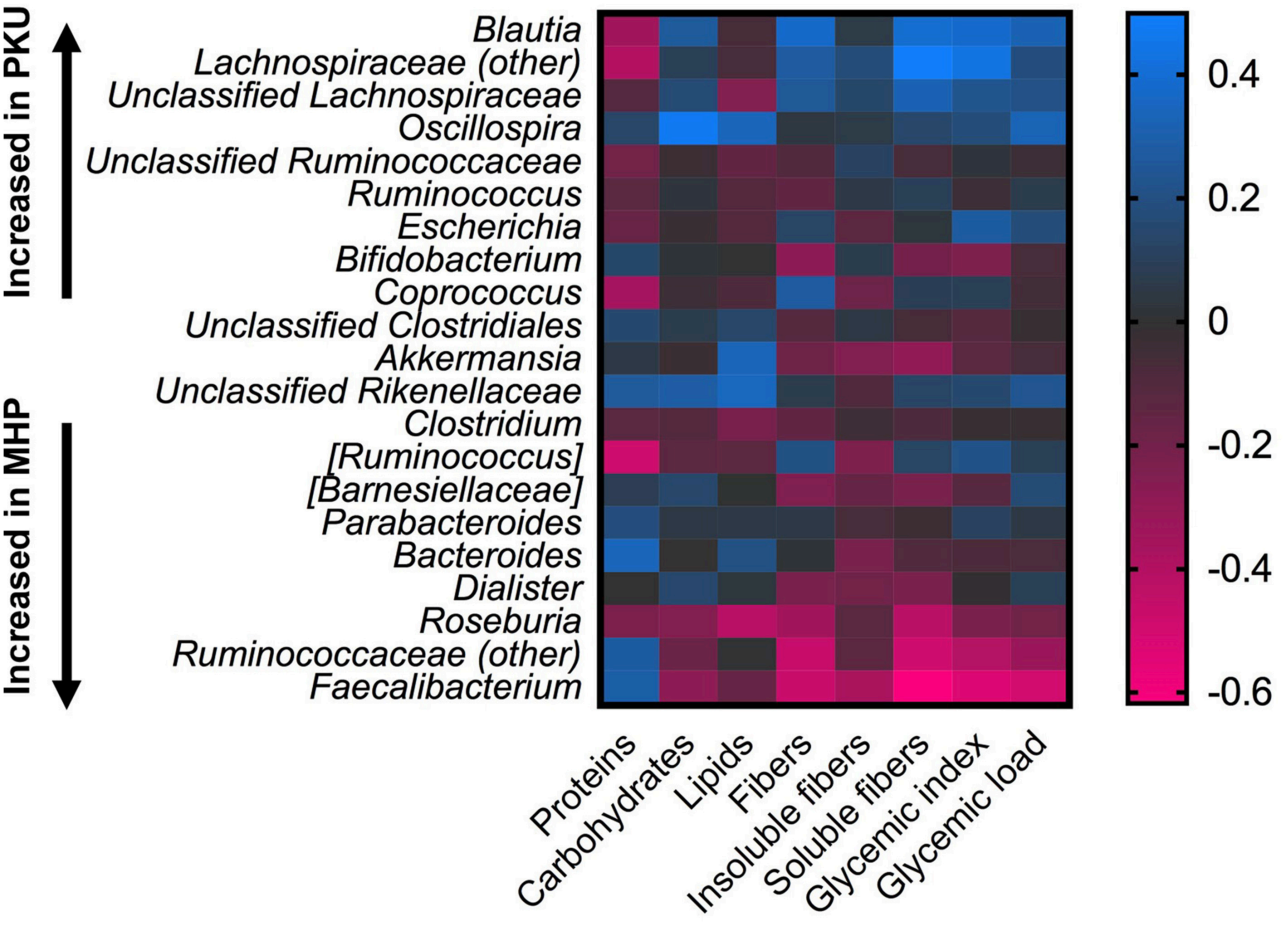
Correlations between microbiota and dietary information. Heatmap showing Spearman's correlations between the most abundant microbial genera and nutritional values. Color intensity represents the degree of association; positive correlations are depicted in blue and negative in magenta.

Statistical correlation analysis between diet and microbiota showed that *Faecalibacterium* spp., significantly increased in MHP group, negatively correlated with fiber intake, both soluble and insoluble fibers (*R* = −0.61; *R* = −0.37), and with GI, GL (*R* = −0.53 and *R* = −0.49, respectively). *Ruminococcaceae* family as well as its genus *Ruminococcaceae (other)*, with higher relative abundance among MHP patients (Figure 4), negatively related with GI and soluble fibers (*R* = −0.40 and *R* = −0.49, respectively), while an opposite trend was observed among the *Lachnospiraceae (other)* genus within the same nutritional values (GI *R* = 0.43; soluble fibers *R* = 0.49). *Oscillospira*, slightly higher in PKU children, was positively related to energy assumption and carbohydrates (*R* = 0.43 and *R* = 0.47, respectively); *Roseburia*, genus enriched in MHP, was found negatively related to soluble fibers (*R* = −0.42) and to lipids (*R* = −0.42). All of these correlation coefficients were statistically significant (*p* < 0.05).

### SCFAs and Gut Microbiota Correlation

As reported in our previous work (Verduci et al., 2018), a decrease in total SCFAs and in particular in butyrate production was observed in PKU children compared with MHP children (*p* = 0.044 and *p* = 0.026, respectively). Although not statistically significant, acetate (*p* = 0.161) was also reduced in PKU group. Propionate and the isoforms iso-butyrate and iso-valerate, instead, were similar.

Correlation analysis with gut microbiota composition revealed several significant interactions between the relative abundance of certain taxa and fecal SCFA concentrations.

Acetate was found negatively related to *Coprococcus* (*R* = −0.42) and to *Blautia* (*R* = −0.50), genera enriched in PKU. Butyrate was found inversely correlated to *Lachnospiraceae (other)* (*R* = −0.41). Propionate was found to positively correlate to *Bacteroides* (*R* = 0.49) and inversely to *Unclassified Lachnospiraceae* (*R* = −0.42) and to *Blautia* (*R* = −0.56). The branched-chain fatty acids iso-butyrate and iso-valerate were negatively related to *Unclassified Lachnospiraceae* (*R* = −0.64, *R* = −0.65, respectively), to *Clostridium* spp. (*R* = −0.45 and *R* = −0.46) and to *Blautia* spp (*R* = −0.40, only iso-butyrate) and positively correlated to *Akkermansia* (*R* = 0.47 and *R* = 0.44). All correlation coefficients were statistically significant (*p* < 0.05).

### Fecal Calprotectin Concentrations in PKU and MHP Patients

To investigate whether PKU subjects are chronically inflamed in the gut, we quantified fecal calprotectin, a recognized biomarker for gastrointestinal diseases (Pang et al., 2014). No significant differences were recorded between PKU and MHP groups (24.8 ± 14.9 μg/g and 40.6 ± 28.3 μg/g, *p* = 0.10, respectively).

## Conclusions and Discussion

The aim of this study was to investigate the impact of a low-Phe diet on microbial gut community and its possible consequences on PKU patient wellbeing. Indeed, according to European guidelines for PKU management, the recommended diet should start as early as possible, usually before the age of 10 days, to prevent neurological damage (Singh et al., 2014; van Spronsen et al., 2017). This age corresponds to a well-recognized crucial step in microbiota acquisition and maturation (Dominguez-Bello et al., 2019) on which environmental factors may have a profound impact.

To rule out a direct effect of PAH deficiency in microbiota alteration, subjects with mild hyperphenylalaninemia, under normal diet, were enrolled as control group.

Bioinformatic analyses revealed several changes in the microbial taxa inhabiting the PKU gut compared with MHP subjects, as already suggested by Pinheiro de Oliveira et al. (2016). Nevertheless, some differences were observed between their study and ours, mainly ascribable to the different enrolled control group (healthy children instead of MHP), the different age range (4 out of their 8 PKU patients were <2 years-old), the sequencing method used (Ion Torrent vs. Illumina) and the subjects' ethnicity (Brazilian vs. Italians).

In our cohort, the relative abundance of Firmicutes and Bacteroidetes was similar, with slight decrease of both phyla in the PKU group. Although not statistically significant, both *Bacteroides* and *Prevotella*, the two main genera belonging to the Bacteroidetes, were more relatively abundant in MHP children. While *Bacteroides* result is not surprising, probably related to the reduced protein intake (David et al., 2014) in Phe-free diet, *Prevotella* is usually associated with increased fiber intake (De Filippo et al., 2010), typical of the PKU diet. Similarly, the *Prevotella/Bacteroides* ratio was slightly higher in MHP group, in contrast to the common finding of a higher ratio in strict vegetarians/vegans (Wu et al., 2011; Franco-de-Moraes et al., 2017). Recently, a high *Prevotella/Bacteroides* ratio has been suggested to be associated with an improvement of glucose response, possibly preventing cardiometabolic diseases (Sandberg et al., 2018).

The most relevant shifts, however, concerned the Firmicutes phylum. Indeed, the PKU gut microbiota was enriched in *Blautia* spp. and *Clostridium* spp. and depleted in *Faecalibacterium* spp., as anticipated in our previous work by absolute real-time PCR quantification (Verduci et al., 2018). As discussed, it was reasonable to expect that the higher fiber intake in PKU patients would have increased *Faecalibacterium* spp. proliferation, but the relative abundance of this genus showed an opposite trend. However, as described by Benus et al. (2010), a fiber-supplemented diet does not necessarily increase *F. prausnitzii* and *Ruminococcus* populations compared with a normal balanced diet. Moreover, the quality of fibers (Verduci et al., 2018) as well as the supplementation of some PKU special low protein products with inulin could also impact the abundance of these genera.

It is intriguing that the indicator species analysis showed an association between MHP phenotype and *F. prausnitzii*, a known biomarker for health status (Lopez-Siles et al., 2017). In healthy adults, this bacterial genus commonly represents more than 5% of the total gut bacterial population (Miquel et al., 2013) and is one of the major butyrate producers. Butyrate is considered the main energy source of colonocytes and displays anti-inflammatory properties in the colonic mucosa (Flint et al., 2012). *A. muciniphila* also characterized the MHP gut microbiota in the indicator species analysis. *A. muciniphila*, a mucin-degrading bacterium, is considered a potential probiotic, that is able to maintain intestinal integrity (Zhai et al., 2018). As already reported by other authors (Arumugam et al., 2011; Cani and de Vos, 2017), *A. muciniphila* is positively associated with members of the family *Ruminococcaceae*, probably sharing nutritional requirements or cross feeding phenomena.

In contrast, PKU microbial communities were characterized by *Ruminococcus bromii*, a well-known starch degrader that belongs to the non-butyrate-forming *Ruminococcaceae* (Ze et al., 2012; Kettle et al., 2015).

Overall, in agreement with the lower total fecal SCFAs content and in particular of butyrate, the PKU gut microbiota was depleted in butyrate-producing species and enriched in genera, i.e., *Blautia*, that are recognized to exert a pro-inflammatory effect on gut mucosa. Indeed, *Blautia* spp. has been demonstrated to induce cytokines secretion, like tumor necrosis factor alpha (TNF-alpha), involved in immune acute phase response (Tuovinen et al., 2013).

Moreover, about half of PKU subjects were characterized by an increase in the relative abundance of Proteobacteria, in particular *Escherichia* spp. Proteobacteria is a phylum of Gram-negative microorganisms whose membrane lipopolysaccharide is a well-known inducer of innate immune responses (Hotamisligil, 2006).

As discussed for the phyla Firmicutes and Bacteroidetes, the enrichment in Proteobacteria observed in PKU children is in contrast with the reported underrepresentation of this phylum in children with rural diet, more similar to PKU vegetarian-vegan diet than MHP, compared with children under Western-diet (De Filippo et al., 2010).

Although our study did not show any alteration in calprotectin, a recognized gut inflammation marker, previous work by our group (Moretti et al., 2017) demonstrated an increase in the triglyceride glucose index (TyG index) in PKU children compared with age- and sex-matched healthy controls. TyG index is considered a marker of low-grade inflammation and of peripheral insulin resistance (Er et al., 2016). Moretti et al. (2017) showed in PKU a positive correlation of TyG index with glycemic load, reinforcing a possible link between carbohydrate quality and metabolic disorder predisposition. Indeed, PKU children showed an increased consumption of fast-absorbing carbohydrates that escape gut microbiota fermentation (Verduci et al., 2018), resulting in higher GI and GL.

It is important to note, we also found a divergent response of the Firmicutes phylum with respect to daily GI and GL. Most members of the *Lachnospiraceae* family, with the exception of *Roseburia*, were positively correlated with both indexes (with *Blautia* as the most relevant genus), whereas members of *Ruminococcaceae* family were negatively, in particular *F. prausnitzii*.

The quality of carbohydrates ingested by PKU children might directly affect *Faecalibacterium* abundance. In accordance with our data, Fava and colleagues (Fava et al., 2013) showed that a diet enriched in carbohydrates with a high glycemic index resulted in a decreased *F. prausnitzii* abundance in subjects at-risk of developing a metabolic syndrome. A plausible explanation is that fast-absorbing carbohydrates do not represent a suitable substrate for *F. prausnitzii* growth, commonly fermenting complex carbohydrates. On the other hand, short chain carbohydrates represent a good substrate for *Blautia* spp. (Egshatyan et al., 2016), which were more relatively abundant in the PKU group. The *Blautia* genus encompasses a huge number of strains with different metabolic capabilities (Eren et al., 2015), several of them considered to be acetogens. In contrast, we found an inverse correlation with fecal acetate concentration. However, this observation is in agreement with recent findings by Org et al. (2017) that investigated the relationship between the gut microbiota composition and metabolic disorder traits. The authors also suggested a positive correlation between *Blautia* and body mass index. Indeed, PKU patients, more than MHP subjects, are at risk for excessive weight gain (Scaglioni et al., 2004; Rocha et al., 2013; Couce et al., 2018) and insulin resistance (Moretti et al., 2017; Couce et al., 2018).

The enrichment of PKU low-protein products in simple sugars, is a consequence of their poor palatability. In the last few years, the increasing awareness of the health consequences of PKU diet led to the development of new products that could implement or complement these formulas. For example, some companies have made commercially available products based on glycomacropeptide (GMP). GMP is a protein derived from cheese whey, rich in specific essential amino acids and lacking aromatic amino acids (i.e., Phe). GMP has been recently demonstrated to have prebiotic properties with beneficial effects on the gut microbiota (Sawin et al., 2015).

In addition, new formulas enriched in prolonged-release amino acids have been recently released. Such medical foods, meant to allow a physiological-like adsorption, are strongly improved in palatability thanks to the amino acids coating layer (Giarratana et al., 2018). It still remains to be evaluated whether an improvement in current free-amino acid formulas, as well as increased attention to the management of dietary carbohydrate quality in PKU diet (with a particular focus on special low protein products), can rebalance the PKU microbial community.

## Ethics Statement

The study was approved by the ethics committee (Comitato Etico Milano Area 1, Protocol number 2015/ST/135). The parents of eligible children or their legal guardian received a detailed explanation of the study and signed a consent form.

## Author Contributions

EB and EV designed the study. GB, FB, and CC performed experiments and data analysis, and drafted the manuscript. CC and MS performed microbiota data analysis. VR performed subject enrollment and analyzed clinical data. EB, EV, and GM performed supervision and writing—review and editing.

### Conflict of Interest Statement

The authors declare that the research was conducted in the absence of any commercial or financial relationships that could be construed as a potential conflict of interest.

## Supplementary Material

Raw reads are available in NCBI Short Read Archive (SRA, http://www.ncbi.nlm.nih.gov/sra) under accession number PRJNA447916.
