# Supplementary material for: Phenylketonuria Diet Promotes Shifts in Firmicutes Populations
Source: Front Cell Infect Microbiol. 2019 Apr 16;9:101. doi: 10.3389/fcimb.2019.00101 (PMC6477998; doi:10.3389/fcimb.2019.00101)
Supplement: Supplementary file 1 [file Table_1.docx]

**Table S1. Gut microbiota composition in MHP and PKU children.** Major bacterial groups subdivided among three phylogenetic levels (phylum, family, genus), reported as average relative abundance and standard deviation. P-values <0.05 were considered significant.

| **TAXONOMIC LEVEL** | | | | | **MHP** | **PKU** | | | **p-value** | | | |
| --- | --- | --- | --- | --- | --- | --- | --- | --- | --- | --- | --- | --- |
| **Phylum** | **Family** | **Genus** | | |  | | | | | | |  |
| FIRMICUTES |  |  | | 57.7 ± 18.3 | | | 59.1 ± 15.5 | | 0.843 |  | |  |
|  | *Ruminococcaceae* |  | 29.5 ± 11.2 | | | | 27.0 ± 10.7 | | 0.553 |  | |  |
|  |  | *Faecalibacterium* | 8.7 ± 4.3 | | | | 4.52 ± 4.1 | | 0.001 | ******* | |  |
|  |  | *Ruminococcus* | 4.5 ± 4.3 | | | | 6.2 ± 5.6 | | 0.514 |  | |  |
|  |  | *Oscillospira* | 2.6 ± 1.8 | | | | 3.8 ± 3.6 | | 0.638 |  | |  |
|  |  | *Ruminococcaceae (other)* | 7.1± 8.3 | | | | 2.8 ± 4.2 | | 0.03 | ***** | |  |
|  |  | *Unclass. Ruminococcaceae* | 6.13 ± 5.36 | | | | 9.28 ± 8.86 | | 0.514 |  | |  |
|  | *Lachnospiraceae* |  | 17.3 ± 10.0 | | | 23.0 ± 12.2 | | 0.149 | | |  | |
|  |  | *Roseburia* | 5.4 ± 5.9 | | | | 4.37 ± 5.7 | | 0.457 |  | |  |
|  |  | *Coprococcus* | 2.0 ± 2.2 | | | | 2.0 ± 1.6 | | 0.438 |  | |  |
|  |  | *Clostridium* | 0.2 ± 0.3 | | | | 1.9 ± 2.6 | | 0.002 | ****** | |  |
|  |  | *Blautia* | 1.1 ± 0.9 | | | | 2.4 ± 2.0 | | 0.004 | ****** | |  |
|  |  | *[Ruminococcus]* | 0.9 ± 1.0 | | | | 2.9 ± 4.5 | | 0.035 | ***** | |  |
|  |  | *Lachnospiraceae (other)* | 0.4 ± 0.3 | | | | 1.8 ± 2.5 | | 0.019 | ***** | |  |
|  |  | *Unclass.Lachnospiraceae* | 5.2 ± 6.8 | | | | 6.5 ± 4.9 | | 0.104 |  | |  |
|  | *Veillonellaceae* |  | 4.8 ± 4.1 | | | | 1.6 ± 2.1 | | 0.002 | ****** | |  |
|  |  | *Dialister* | 3.3 ± 3.8 | | | | 0.7 ± 2.1 | | 0.036 | ***** | |  |
|  | *Clostridiaceae* |  | 1.3 ± 1.4 | | | | 2.0 ± 3.7 | | 0.964 |  | |  |
|  |  | *Clostridium* | 0.6 ± 0.7 | | | | 1.7± 3.4 | | 0.939 |  | |  |
|  | *Unclassified Clostridiales* |  | 2.4 ± 3.5 | | | | 3.2 ± 8.0 | | 0.843 |  | |  |
|  | *Streptococcaceae* |  | 1.1 ± 2.6 | | | | 0.5 ± 0.6 | | 0.553 |  | |  |
|  |  | *Streptococcus* | 1.2 ± 2.6 | | | | 0.5 ± 0.6 | | 0.553 |  | |  |
|  | *Erysipelotrichaceae* |  | 0.3 ± 0.3 | | | | 0.4 ± 0.2 | | 0.028 | ***** | |  |
|  | *Christensenellaceae* |  | 0.1 ± 0.1 | | | | 0.5 ± 0.7 | | 0.149 |  | |  |
|  | *Lactobacillaceae* |  | 0.3 ± 0.9 | | | | 0.0 ± 0.0 | | 0.152 |  | |  |
| BACTEROIDETES |  |  | 32.3 ± 15.8 | | | | 26.1 ± 17.3 | | 0.186 |  | |  |
|  | *Bacteroidaceae* |  | 26.1 ± 12.9 | | | | 21.8 ± 15.3 | | 0.267 |  | |  |
|  |  | *Bacteroides* | 26.1 ± 12.9 | | | | 21.8 ± 15.3 | | 0.267 |  | |  |
|  | *Rikenellaceae* |  | 2.0 ± 1.4 | | | | 2.3 ± 2.0 | | 0.915 |  | |  |
|  |  | *Unclas.Rikenellaceae* | 2.0 ± 1.4 | | | | 2.2 ± 2.0 | | 0.939 |  | |  |
|  | *Prevotellaceae* |  | 2.0 ± 8.7 | | | | 0.4 ± 0.9 | | 0.338 |  | |  |
|  |  | *Prevotella* | 2.0 ± 8.7 | | | | 0.4 ± 0.9 | | 0.338 |  | |  |
|  | *Porphyromonadaceae* |  | 1.2 ± 0.9 | | | | 1.0 ± 0.8 | | 0.638 |  | |  |
|  |  | *Parabacteroides* | 1.1 ± 0.9 | | | | 1.0 ± 0.9 | | 0.66 |  | |  |
| VERRUCOMICROBIA |  |  | 5.9 ± 13.3 | | | | 3.4 ± 6.7 | | 0.254 |  | |  |
|  | *Verrucomicrobiaceae* |  | 5.9 ± 13.3 | | | | 3.4 ± 6.7 | | 0.254 |  | |  |
|  |  | *Akkermansia* | 5.9 ± 13.3 | | | | 3.4 ± 6.7 | | 0.254 |  | |  |
| ACTINOBACTERIA |  |  | 2.5 ± 2.4 | | | | 6.8 ± 14.0 | | 0.533 |  | |  |
|  | *Bifidobacteriaceae* |  | 2.3 ± 2.4 | | | | 6.7 ± 14.0 | | 0.533 |  | |  |
|  |  | *Bifidobacterium* | 2.3 ± 2.40 | | | | 6.7 ± 14.0 | | 0.514 |  | |  |
| PROTEOBACTERIA |  |  | 1.4 ± 1.0 | | | | 4.0 ± 5.6 | | 0.727 |  | |  |
|  | *Enterobacteriaceae* |  | 0.7 ± 0.9 | | | | 3.5 ± 5.4 | | 0.294 |  | |  |
|  |  | *Escherichia* | 0.7 ± 0.9 | | | | 2.8 ± 5.1 | | 0.37 |  | |  |
| TM7 |  |  | 0.7 ± 0.0 | | | | 0.3 ± 0.5 | | 0.003 | ****** | |  |
|  | *Unclassified TM7-3* |  | 0.0 ± 0.0 | | | | 0.2 ± 0.4 | | 0.002 | ****** | |  |
