# Supplementary material for: Phenylketonuria Diet Promotes Shifts in Firmicutes Populations
Source: Front Cell Infect Microbiol. 2019 Apr 16;9:101. doi: 10.3389/fcimb.2019.00101 (PMC6477998; doi:10.3389/fcimb.2019.00101)
Supplement: Supplementary file 2 [file Table_2.docx]

**Table S2. Gut microbiota correlation patterns.** Genera co-occurrence matrix of the studied cohort. Only significant R values, indicating positive and negative correlations using Spearman’s rank, of the most abundant genera are reported. Blank spaces (--) represent non significant correlations. (p>0.05).

*Unclass. Clostridiales*

*Ruminococcaceae (other)*

*Unclass. Lachnospiraceae*

*Unclass. Rikenellaceae*

*Faecalibacterium*

*Oscillospira*

*[Barnesiellaceae]*

*Clostridium*

*Blautia*

*[Ruminococcus]*

*Coprococcus*

*Bifidobacterium*

*Akkermansia*

*Roseburia*

*Bacteroides*

|  | *Bacteroides* | | *Faecalibacterium* | |  | |  | |  | | |  | |  | |  | |  | |  | |  | | |  | |  | |  | *Lachnospiraceae (other)* | *Parabacteroides* |  |
| --- | --- | --- | --- | --- | --- | --- | --- | --- | --- | --- | --- | --- | --- | --- | --- | --- | --- | --- | --- | --- | --- | --- | --- | --- | --- | --- | --- | --- | --- | --- | --- | --- |
| *Bacteroides* | 1 | | -- | | -- | | -- | | -0.37 | | | -- | | -- | | -- | | -- | | 0.55 | | -- | | | -- | | -0.35 | | -- | -- | -- | -- |
| *Faecalibacterium* | -- | | 1 | | -- | | 0.42 | | -- | | | -- | | -- | | -- | | -- | | -- | | -- | | | -- | | -- | | -- | -- | -- | -- |
| *Unclass. Lachnospiraceae* | -- | | -- | | 1 | | -- | | -- | | | -0.62 | | -0.35 | | -- | | -- | | -- | | -- | | | -- | | 0.33 | | -- | -- |  | -- |
| *Ruminococcaceae (other)* | -- | | 0.42 | | -- | | 1 | | 0.38 | | | -- | | -- | | -- | | -- | | -- | | -- | | | -- | | -- | | -- | -0.50 | -- | -- |
| *Roseburia* | -0.37 | | -- | | -- | | 0.38 | | 1 | | | -- | | -- | | -0.36 | | -- | | -- | | -- | | | -- | | -- | | 0.57 | -- | -- | -- |
| *Akkermansia* | -- | | -- | | -0.62 | | -- | | -- | | | 1 | | -- | | 0.57 | | 0.35 | | -- | | -- | | | -- | | -0.41 | | -- | -0.35 | -- | -- |
| *Bifidobacterium* | -- | | -- | | -0.35 | | -- | | -- | | | -- | | 1 | | -- | | -- | | -- | | -- | | | -- | | -- | | -- | -- | -- | -- |
| *Oscillospira* | -- | | -- | | -- | | -- | | -0.36 | | | 0.57 | | -- | | 1 | | 0.46 | | 0.61 | | -- | | | -0.33 | | -- | | -0.48 | -- | -- | -- |
| *Unclass. Clostridiales* | -- | | -- | | -- | | -- | | -- | | | 0.35 | | -- | | 0.46 | | 1 | | -- | | -0.34 | | | -- | | -- | | -- | -- | -- | 0.38 |
| *Unclass. Rikenellaceae* | 0.55 | | -- | | -- | | -- | | -- | | | -- | | -- | | 0.61 | | -- | | 1 | | -0.37 | | | -0.40 | | -- | | -0.34 | -- | 0.33 | -- |
| *Coprococcus* | -- | | -- | | -- | | -- | | -- | | | -- | | -- | | -- | | -0.34 | | -0.37 | | 1 | | | -- | | 0.6 | | -- | -- | -- | -- |
| *[Ruminococcus]* | -- | | -- | | -- | | -- | | -- | | | -- | | -- | | -0.33 | | -- | | -0.40 | | -- | | | 1 | | 0.67 | | -- | 0.39 | -0.41 | -- |
| *Blautia* | -0.355 | | -- | | 0.33 | | -- | | -- | | | -0.41 | | -- | | -- | | -- | | -- | | 0.36 | | | 0.67 | | 1 | | -- | 0.45 | -0.45 | -- |
| *Clostridium* | -- | | -- | | -- | | -- | | 0.57 | | | -- | | -- | | -0.48 | | -- | | -0.34 | | -- | | | -- | | -- | | 1 | -- | -0.42 | -- |
| *Lachnospiraceae (other)* | -- | | -- | | -- | | -0.50 | | -- | | | -0.35 | | -- | | -- | | -- | |  | | -- | | | 0.39 | | 0.45 | | -- | 1 | -- | -0.47 |
| *Parabacteroides* | 0.426 | | -- | | -- | | -- | | -- | | | -- | | -- | | -- | | -- | | 0.33 | | -- | | | -0.41 | | -0.45 | | -0.42 | -- | 1 | 0.43 |
| *[Barnesiellaceae]* | -- | | -- | | -- | | -- | | -- | | | -- | | -- | | -- | | 0.38 | | -- | | -- | | | -- | | -- | | -- | -0.47 | 0.43 | 1 |
|  |  |  | |  | |  | |  | |  |  | |  | |  | |  | |  | |  | |  |  | |  | |  |  |  |  |  |
